# Diaphragm ultrasound evaluation during weaning from mechanical ventilation in COVID-19 patients: a pragmatic, cross-section, multicenter study

**DOI:** 10.1186/s12931-022-02138-y

**Published:** 2022-08-21

**Authors:** Luigi Vetrugno, Daniele Orso, Francesco Corradi, Gianluca Zani, Savino Spadaro, Francesco Meroi, Natascia D’Andrea, Tiziana Bove, Gianmaria Cammarota, Edoardo De Robertis, Samuele Ferrari, Marcello Guarnieri, Margherita Ajuti, Maurizio Fusari, Domenico Luca Grieco, Cristian Deana, Enrico Boero, Federico Franchi, Sabino Scolletta, Salvatore Maurizio Maggiore, Francesco Forfori

**Affiliations:** 1 Department of Medical, Oral and Biotechnological Sciences, University of Chieti-Pescara, Chieti, Italy; 2 Department of Anesthesiology, Critical Care Medicine and Emergency, SS. Annunziata Hospital, Via dei Vestini, 66100 Chieti, Italy; 3 Department of Medicine (DAME), University of Udine, Udine, Italy; 4 Department of Anesthesia and Intensive Care Medicine, ASUFC University Hospital of Udine, Udine, Italy; 5 Department of Surgical, Medical, Molecular Pathology and Critical Care Medicine, University of Pisa, Pisa, Italy; 6 Department of Anesthesia and Intensive Care, Ravenna, Italy; 7 Department of Morphology, Surgery and Experimental Medicine, Intensive Care Unit, University of Ferrara, Sant’Anna Hospital, Ferrara, Italy; 8 Department of Medicine and Surgery, Università degli Studi di Perugia, Perugia, Italy; 9 Anestesia and Intensive Care Service 2, Azienda Ospedaliera di Perugia, Perugia, Italy; 10 Department of Anesthesia and Intensive Care, Niguarda Hospital, Piazza dell’Ospedale Maggiore 3, 20162 Milano, Italy; 11 Intensive Care Unit, Fondazione Policlinico Universitario A. Gemelli IRCCS, Rome, Italy; 12 Dipartimento di Scienze Chirurgiche, Università degli Studi di Torino, Turin, Italy; 13 Department of Medicine, Surgery and Neuroscience, Anesthesia and Intensive Care Unit, University of Siena, Siena, Italy; 14 Department of Innovative Technologies in Medicine and Dentistry, Gabriele d’Annunzio University of Chieti-Pescara, Chieti, Italy

**Keywords:** Diaphragm, COVID-19, Mechanical ventilation, Ultrasound, Weaning failure

## Abstract

**Background:**

Diaphragmatic dysfunction is a major factor responsible for weaning failure in patients that underwent prolonged invasive mechanical ventilation for acute severe respiratory failure from COVID-19. This study hypothesizes that ultrasound measured diaphragmatic thickening fraction (DTF) could provide corroborating information for weaning COVID-19 patients from mechanical ventilation.

**Methods:**

This was an observational, pragmatic, cross-section, multicenter study in 6 Italian intensive care units. DTF was assessed in COVID-19 patients undergoing weaning from mechanical ventilation from 1st March 2020 to 30th June 2021. Primary aim was to evaluate whether DTF is a predictive factor for weaning failure.

**Results:**

Fifty-seven patients were enrolled, 25 patients failed spontaneous breathing trial (44%). Median length of invasive ventilation was 14 days (IQR 7–22). Median DTF within 24 h since the start of weaning was 28% (IQR 22–39%), RASS score (− 2 vs − 2; p = 0.031); Kelly-Matthay score (2 vs 1; p = 0.002); inspiratory oxygen fraction (0.45 vs 0.40; p = 0.033). PaO_2_/FiO_2_ ratio was lower (176 vs 241; p = 0.032) and length of intensive care stay was longer (27 vs 16.5 days; p = 0.025) in patients who failed weaning. The generalized linear regression model did not select any variables that could predict weaning failure. DTF was correlated with pH (RR 1.56 × 10^27^; p = 0.002); Kelly-Matthay score (RR 353; p < 0.001); RASS (RR 2.11; p = 0.003); PaO_2_/FiO_2_ ratio (RR 1.03; p = 0.05); SAPS2 (RR 0.71; p = 0.005); hospital and ICU length of stay (RR 1.22 and 0.79, respectively; p < 0.001 and p = 0.004).

**Conclusions:**

DTF in COVID-19 patients was not predictive of weaning failure from mechanical ventilation, and larger studies are needed to evaluate it in clinical practice further.

*Registered:* ClinicalTrial.gov (NCT05019313, 24 August 2021).

## Background

Interstitial pneumonia and adult respiratory distress syndrome (ARDS) are severe COVID-19 complications that lead to intensive care unit (ICU) admission. Mortality remains higher than 30%, especially in non-vaccinated individuals that develop severe ARDS [1–3]. Initial COVID-19 interstitial pneumonia primarily shows alveolar-capillary shunting without clinically relevant respiratory signs (silent hypoxemia). However, as the infection progresses, so does the involvement of lung parenchyma, outlining a more severe clinical picture (manifest hypoxemia). It includes increasing elastance, right-left shunting and lung de-recruitment ultimately requiring intensive care admission, sedation, pronation, and muscle relaxation to reach adequate gas exchanges [4]. These patients require prolonged mechanical ventilation, often longer than 2–3 weeks, potentially inducing diaphragmatic dysfunction that could impact ventilatory support duration and weaning outcome [5]. Ultrasounds represent a non-invasive bedside approach to evaluate diaphragmatic thickening. This field has rapidly grown in recent years, with many publications concerning non-COVID-19 patients. The expression of transdiaphragmatic pressure, in terms of diaphragmatic thickening fraction (DTF), indicates muscular contractility. Goligher et al. first described the relationship between changes in transdiaphragmatic pressure and DTF in healthy subjects [6]. Diaphragmatic dysfunction had already been documented before a possible correlation between diaphragmatic dysfunction and weaning failure from mechanical ventilation emerged in patients with COVID-19 pneumonia. Although it is known that diaphragmatic force is mainly preserved or improved in the first phases of COVID-19 infection, some patients have shown early dysfunction in spontaneous breathing [7–9]. However, only few studies focused on diaphragmatic function during weaning from mechanical ventilation in these patients. This study evaluates the role of diaphragmatic dysfunction, as assessed by DTF at the beginning of weaning from mechanical ventilation, and weaning failure in COVID-19 patients. The aim of this study was that the DTF value could be a potential predictive factor of weaning success.

## Methods

This was an observational, pragmatic, cross-section, multicenter study. The study was conducted in 6 Italian ICUs as Udine, Ferrara, Ravenna, Pisa, Turin and Milan, from 1st March 2020 to 30th June 2021. Every center’s Ethical Committee Board approved the study following local guidelines for ethical standards and good clinical practice. Each principal investigator was responsible for obtaining informed consent, in accordance with hospital privacy protocols during the emergency, local rules, and institutional regulations. All ethic committees’ approvals are added in the dedicated section at the end of the article. The study was registered at ClinicalTrial.gov (NCT05019313, 24 August 2021) and observed the STROBE guidelines for an observational study.

Inclusion criteria were as follows: age > 18 years, admission to a COVID-19 ICU with hypoxemic respiratory failure (defined as PaO_2_ < 70 mmHg or PaO_2_/FiO_2_ < 150), start of weaning from mechanical ventilation, defined as the ability to maintain respiratory stability (no dyspnea, respiratory rate < 25/min, percutaneous oxygen saturation (SpO_2_) ≥ 96%) with mechanical ventilation in pressure support mode at standardized settings (pressure support of 8 cmH_2_O, PEEP 5 cmH_2_O, and inspired fraction of oxygen (FiO_2_) < 0.5). Exclusion criteria were: being non-cooperative (Kelly-Matthay score ≥ 5) or Richmond Agitation-Sedation Scale (RASS) ≥  + 2, participation refusal, tracheostomy, unstable clinical conditions or multiple organ failure (> 2 organs). Weaning failure was defined as failure to pass the spontaneous-breathing trial or the need for re-intubation within 48 h following extubation. [10, 11] DTF was evaluated within 24 h since the start of weaning. DTF was assessed for the right hemidiaphragm, using a linear probe to identify a muscular layer between two hyperechoic lines, the pleura and peritoneum, superficial to the liver, with patients in supine position with the trunk elevated 10°–15°. In all patients, DTF was calculated while patients were ventilated with pressure support ventilation, with standardized settings (8 cmH_2_O pressure support, 5 cmH_2_O positive end-expiratory pressure (PEEP), FiO_2_ < 0.5) and hemodynamic stability. Measurements were obtained by employing instrumentations available in the respective ICUs. DTF was calculated using M-mode imaging, maximal diaphragm thickness during inspiration (Tdi, pi), minus diaphragm thickness at end-expiration (Tdi,ee), divided by Tdi,ee and multiplied by 100. Three consecutive measures were obtained, and the mean value was recorded. We also collected Simplified Acute Physiology Score 2 (SAPS2); RASS; Kelly-Matthay score; the systolic and diastolic arterial pressure; the heart rate; the respiratory rate; the body temperature; the base excess; non-invasive ventilation trial before invasive mechanical ventilation and its duration; invasive mechanical ventilation duration; ICU and hospital length of stay.

### End-points

The primary end-point was to verify if the DTF predicts weaning success/failure from mechanical ventilation. The secondary end-point was to analyze the co-variables correlated with DTF values.

### Sample size estimation

We assumed a priori a prevalence of diaphragmatic dysfunction of 42.5% [12]. To achieve the adequate prevalence within this study with a two-sided 95% confidence interval and a maximum accuracy error of 15%, 38 patients will be needed. However, considering a drop out of 20% due to the COVID-19 environment problem in data acquisition, we estimate to enrol a minimum of 45 subjects.

### Statistical analysis

Described variables were recorded as median [IQR] for continuous variables and frequencies (%) for categorical variables. Study population was divided according to weaning success or failure. After data distribution was verified, group comparison was executed using Mann-Withney test. Chi-squared test or Fisher's exact test were used for categorical variables. A Benjamini & Hochberg correction for the multiplicity of the p-value was considered. P-values ≤ 0.05 were considered statistically significant. A generalized linear regression model was adopted to detect variables correlated with weaning failure. A multivariable linear regression model was applied to identify variables related to DTF. Predictive variables were selected by carrying out a stepwise backward method, which eliminated less predictive variables until the most predictive model was achieved. Further post-hoc sub-analyses were carried out to analyse the behaviour of certain subgroups within the population. Statistical calculations were performed using the R-CRAN platform.

## Results

Fifty-seven patients admitted to participating ICUs from March 2020 to June 2021 were enrolled in the study. The main characteristics of the study population are illustrated in Table 1.

**Table 1:** Study population characteristics and group comparison between patients weaned successfully and unsuccessfully

	Population	Weaning failure	Weaning success	p-value
	N = 57	N = 25	N = 32	
Age [IQR]	65.0 [56.0; 71.0]	64.0 [57.0; 71.0]	66.5 [55.8; 71.0]	0.766
Sex (male, %)	42 (73.7%)	17 (68.0%)	25 (78.1%)	0.577
SAPS2	34.0 [31.0; 41.0]	34.0 [32.0; 41.0]	33.5 [31.0; 41.0]	0.612
RASS	− 1.00 [− 2.00; 0.00]	− 2.00 [− 2.00; − 1.00]	− 1.00 [− 2.00; 0.00]	0.031
KM score	2.00 [1.00;3.00]	2.00 [2.00;3.00]	1.00 [1.00;2.00]	0.002
SAP (mmHg)	133 [123; 149]	130 [123; 140]	140 [124; 151]	0.227
DAP (mmHg)	70 [63; 80]	68 [60; 73]	76 [65; 82]	0.096
HR (bpm)	78 [70; 90]	82 [73; 90]	77 [70; 90]	0.541
RR (bpm)	18 [16; 23]	22 [16; 24]	18 [16; 21]	0.302
Body Temperature (°C)	36.9 [36.5; 37.3]	36.9 [36.7; 37.2]	36.9 [36.5; 37.3]	0.389
SpO_2_ (%)	98 [96; 99]	98 [96; 99]	98 [96; 99]	0.915
FiO_2_	0.40 [0.35; 0.50]	0.45 [0.40; 0.50]	0.40 [0.35; 0.45]	0.033
pH	7.46 [7.43; 7.50]	7.47 [7.42; 7.49]	7.46 [7.43; 7.50]	0.955
PaCO_2_ (mmHg)	43.0 [38.0; 48.0]	43.0 [39.0; 48.0]	41.5 [38.0; 47.2]	0.562
PaO_2_ (mmHg)	85.0 [75.0; 106]	84.0 [72.0; 96.0]	92.0 [79.0; 115]	0.215
PaO_2_/FiO_2_ ratio (mmHg)	216 [164; 280]	176 [144; 231]	241 [183; 308]	0.032
Lactates (mEq/L)	1.10 [0.90; 1.40]	1.10 [0.90; 1.31]	1.10 [0.91; 1.43]	0.981
HCO_3_ (mEq/L)	30.2 [27.9; 32.7]	30.5 [27.9; 32.0]	29.9 [27.7; 32.9]	0.682
BE	6.70 [3.40; 8.90]	7.00 [3.40; 8.10]	6.45 [3.72; 9.00]	0.930
Diam exp. (mm)	1.90 [1.50; 2.50]	1.90 [1.50; 2.50]	1.85 [1.50; 2.52]	0.968
Diam insp. (mm)	2.40 [2.00; 3.20]	2.60 [2.00; 3.60]	2.40 [1.98; 3.20]	0.484
DTF (%)	28.0 [21.7; 38.9]	28.0 [16.7; 57.1]	27.6 [22.1; 37.0]	0.612
Previous NIV	46 (80.7%)	23 (92.0%)	23 (71.9%)	0.090
NIV Length (hours)	15.0 [3.00; 36.0]	20.0 [4.00; 36.0]	7.00 [0.00; 37.0]	0.248
Mech. Vent. duration (days)	14 [7; 22]	15 [8; 24]	13 [7; 22]	0.670
ICU LOS (days)	20.0 [12.0; 31.0]	27.0 [18.0; 37.0]	16.5 [10.0; 27.5]	0.025
Hospital LOS (days)	37.0 [28.0; 47.0]	44.0 [29.0; 51.0]	34.0 [26.8; 43.0]	0.171

*SAPS2* Simplified Acute Physiology Score 2, *RASS* Richmond Agitation-Sedation Scale, *KM score* Kelly–Matthay score, *SAP* Systolic Arterial Pressure, *DAP* diastolic arterial pressure, *HR* heart rate, *RR* Respiratory Rate, *BT* Body Temperature, *BE* Base Excess, *Diam exp.* Expiratory Diaphragmatic Diameter, *Diam insp.* Inspiratory Diaphragmatic Diameter, *DTF* Diaphragmatic Thickening Fraction, *Previous NIV* Non-Invasive Ventilation trial before invasive mechanical ventilation, *NIV Length* NIV trial duration, *Mech. Vent. Length* invasive mechanical ventilation duration, *ICU LOS* ICU Length of Stay, *Hosp LOS* Hospital Length of Stay

Median age was 65 years (IQR 56–71); 42 patients (74%) were male. Forty-six (81%) patients underwent a trial of non-invasive ventilation (NIV) before intubation (duration: 15 h; IQR 3–36). Median length of invasive ventilation was 14 days (IQR 7–22). Median DTF at weaning start (or at SBT start) was 28.0 (IQR 21.7–38.9).

### Comparison between weaning success and failure

**Fig. 1:**
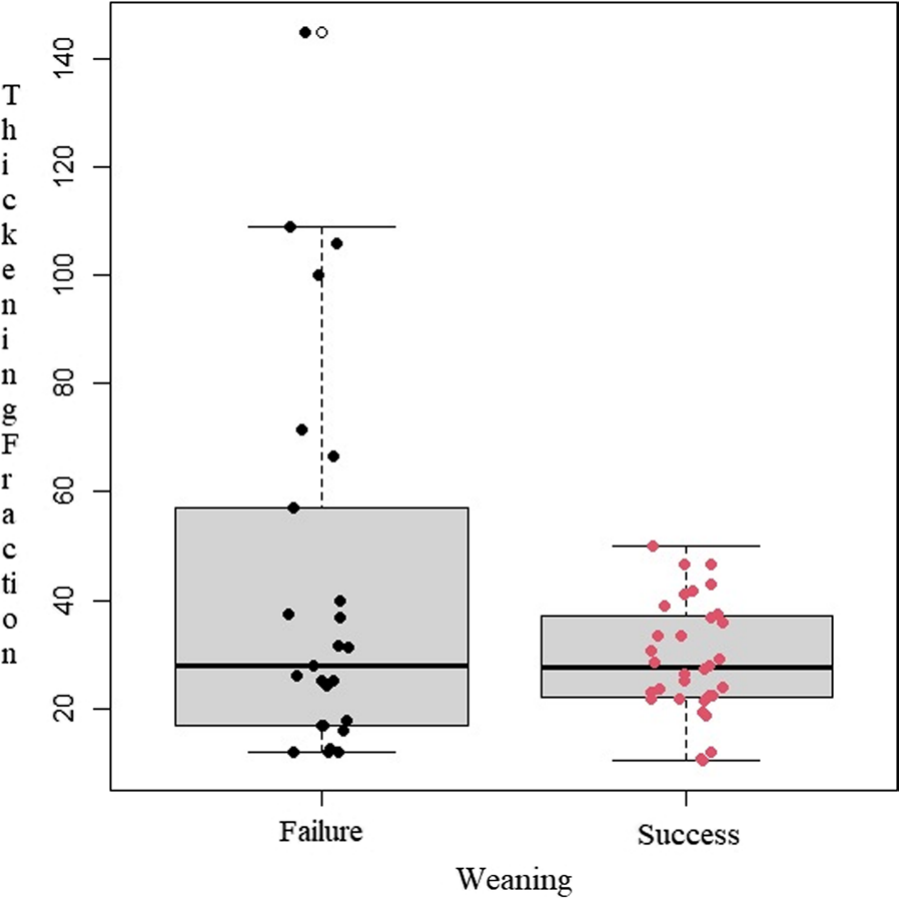
DTF comparison between successfully weaned patients and those who failed weaning from mechanical ventilation. Although DTF medians are not significantly different (28% vs 27.6%; p = 0.612), higher outliers are found in patients who failed weaning

DTF was not different between weaning success and weaning failure groups (Fig. 1).

**Fig. 2:**
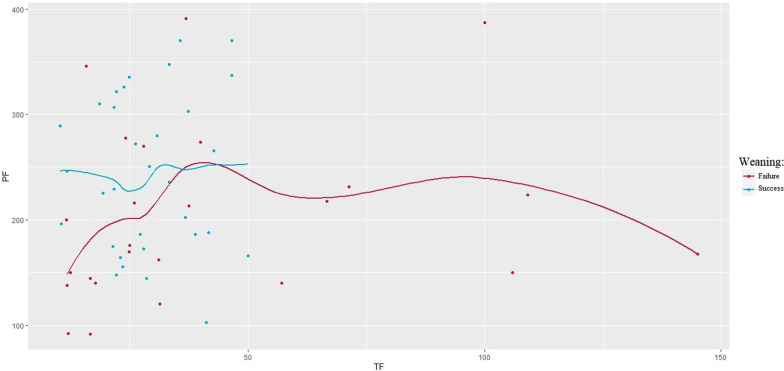
DTF distribution, with respect to PaO_2_/FiO_2_, in successfully weaned patients (in blue) and unsuccessfully (in red). The trend was obtained through a mixed linear regression model, which demonstrates a U-shaped trend in DTF values, lowest PaO_2_/FiO_2_ values at both extremes, for patients who failed weaning

The following parameters were statistically different between groups: RASS (− 2 vs − 2; p = 0.031); Kelly-Matthay score (2 vs 1; p = 0.002); inspiratory oxygen fraction (0.45 vs 0.40; p = 0.033), PaO_2_/FiO_2_ ratio (176 vs 241; p = 0.032) and length of ICU stay (27 vs 16.5 days; p = 0.025) (Table 1). The generalized linear regression model did not identify any variable independently associated with weaning failure. DTF distribution according to PaO_2_/FiO_2_ values in patients who failed weaning showed a rough U-shaped trend, in which extreme DTF values were associated with the lowest PaO_2_/FiO_2_ (Fig. 2).

**Fig. 3:**
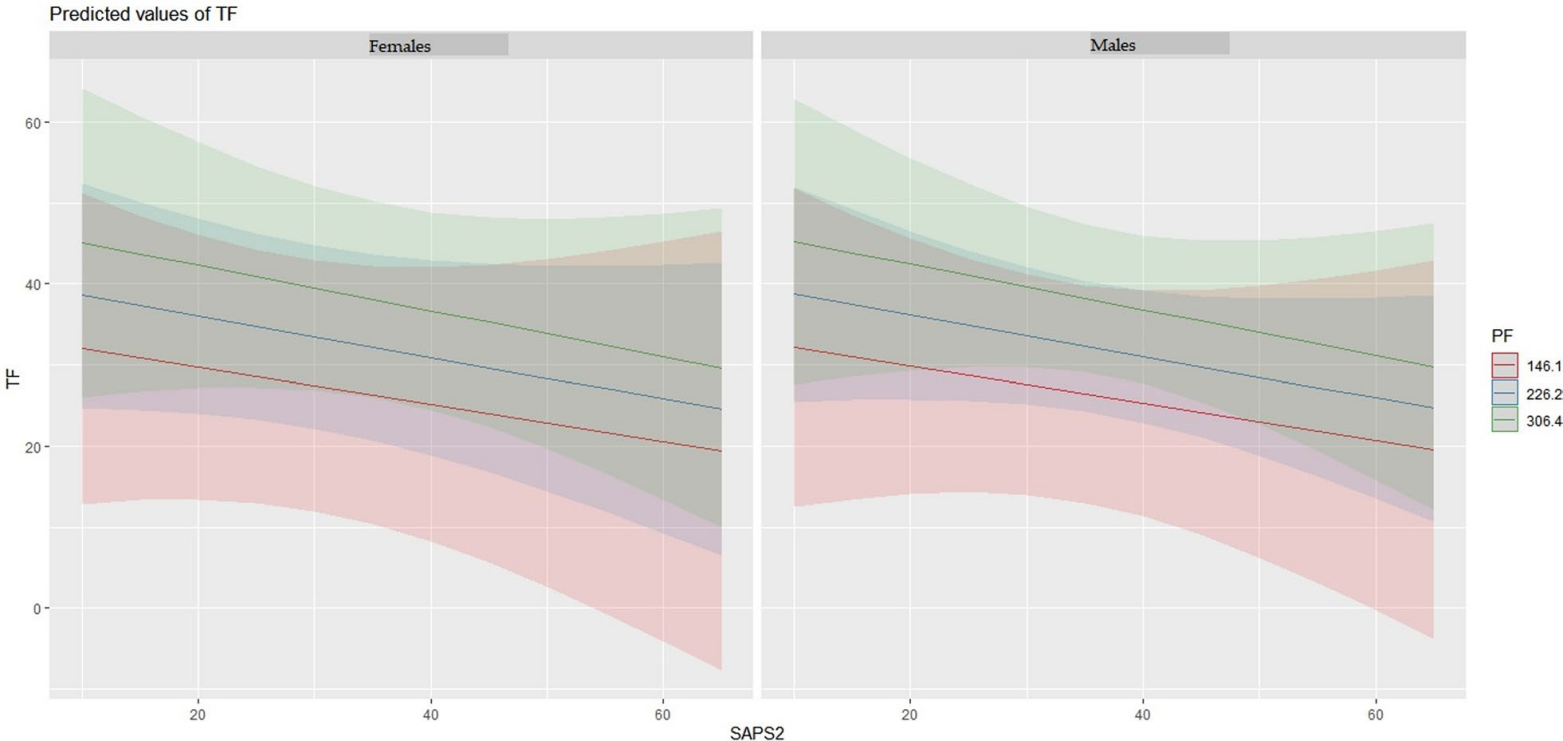
Comparison of SAPS-2 scores (x-axis) and DTF values (y-axis), divided by gender (females on the left and males on the right) and PaO_2_/FiO_2_ ratio subclasses (highest values in green, lowest in red). As shown in the lower right quadrants of the graphs (highest SAPS-2 scores) DTF values are higher in males, compared to females, for each PaO_2_/FiO_2_ subclass

In the regression model performed to identify variables correlated to DTF, pH (RR 1.56 × 10^27^; p = 0.002); Kelly-Matthay score (RR 353; p < 0.001); RASS (RR 2.11; p = 0.003); PaO_2_/FiO_2_ ratio (RR 1.03; p = 0.05); SAPS2 (RR 0.71; p = 0.005); hospital and ICU length of stay (RR 1.22 and 0.79, respectively; p < 0.001 and p = 0.004) were all significantly correlated. Subgroup post-hoc analysis showed that male patients had greater DTF values than female patients, at equal disease severity (SAPS2 score) (Fig. 3).

## Discussion

The study evaluated a possible correlation between weaning failure and DTF values and other potential predictors, in critically ill COVID-19 patients. DTF was found to be unrelated to weaning outcome. In agreement with current literature, the present work highlighted a very high rate of weaning failure, around 40%, requiring longer ICU stay and sedation. Observing gathered data, COVID-19 is likely implicated in determining an initial thickening diaphragm and ultimately lung parenchyma fibrosis [13]. Poulard et al. have stressed how, although DTF ultrasound evaluation has recently become popular, its correlation with transdiaphragmatic pressure (measured by invasive catheters to record esophageal and gastric pressure) is widely variable, especially in patients subjected to mechanical ventilation [14]. It is reasonable to hypothesize that extremes—hypertrophy and atrophy—correlate with a more pronounced respiratory dysfunction [15, 16]. Similarly, ventilatory support, either above or below patient’s needs, might lead to diaphragmatic dysfunction [17]. In addition, patient’s basal diaphragmatic strength could play a determining role in the development of dysfunction, further complicating achievement of a standardized ultrasound method [18, 19].

Other factors related to DTF are degree of sedation (RASS score) and length of hospital stay, which are both linearly implicated. On the contrary, PaO_2_/FiO_2_ shows a very weak correlation. Even though pathophysiological mechanisms of parenchymal damage could be involved, the study data seems to reflect the U-shaped pattern previously highlighted: PaO_2_/FiO_2_ values show a linear correlation with the diaphragm thickening capacity within a range, beyond which at the low end of PaO_2_/FiO_2_, this diaphragm thickening capacity can be almost completely abolished or, on the contrary, excessively expressed (the result of an excess of respiratory effort).

A contributing mechanism to diaphragmatic dysfunction is pronounced respiratory acidosis, which could both be cause and consequence of diaphragmatic thinning; namely, depletion of energy substrates favors a hyperlactatemia that may not be sufficient to support required muscular effort [20]. However, the diaphragm may be the major contributor to lactate production, making the patient prone to acidosis. In our study, pH directly correlated with DTF, a possible expression of respiratory alkalosis. Conversely, the reduction in pH could result from the diaphragmatic metabolic debt condition.

Fever due to viral infection can determine diaphragmatic dysfunction [21] through a “cytokine storm”, which in turn may contribute to muscle catabolism [22]. In a cluster analysis of 55 patients undergoing mechanical ventilation, Vivier et al. detected a linear correlation between PaCO_2_ and DTF [23]. The authors hypothesize that increased DTF results in increased respiratory workload in spontaneously breathing patients. Conversely, the use of sedation, as occurs in patients admitted to ICUs, could lead to muscle deconditioning—similarly to global ICU-acquired weakness—and may be responsible for increased hospital LOS and weaning failure. However, special attention should be paid when labeling diaphragmatic dysfunction as a predictor of ICU-acquired weakness. Jung et al. have shown that, although diaphragmatic dysfunction is frequent in patients with ICU-acquired weakness, about half of patients with the latter condition undergo successful extubation [24].

A recent study by Cammarota et al. found that prone position in spontaneously breathing patients, though supported by non-invasive ventilation, caused an increase in DTF [25]. Authors suggest that the correlation between DTF and ventilation efficacy is more complex than previously thought. The mere evaluation of DTF per se does not account for improvements in ventilation. How far DTF is to be pursued as an expression of greater muscle strength or, vice versa, as a negative expression of increased respiratory workload, has yet to be established. A recent meta-analysis of the diagnostic accuracy of diaphragmatic ultrasound to predict weaning outcome showed low sensitivity [26]. Furthermore, authors highlighted a wide heterogeneity of the studies considered which suggests the extent of the effect of other variables, not directly measured in different subpopulations, can contribute to weaning outcome [27].

Lastly, as hypothesis-generator, the present study shows that male patients, compared to female patients, have higher DTF values for the same severity of disease (SAPS2 score and PaO_2_/FiO_2_ ratio). However, what makes females prone to particularly severe forms of diaphragmatic dysfunction in the course of COVID-19 is yet to be determined.

### Limitations

Study limitations were mainly related to conditions in which it was performed: patient enrollment, data collection and practical methods reflect, in fact, the course of the pandemic. Standardized ventilation criteria before weaning were not established amongst participating centers. Though operators involved in the study are considered experts, different levels of proficiency may result in data collection discrepancies. In order to maximize the inter-observer reproducibility, we have disseminated video tutorials prior the enrollment [28]. Given the pragmatic nature of the study the adopted definition of weaning failure was restricted to an easily applicable and contextualizable definition in all participating centers.

## Conclusions

DTF ultrasound evaluation is a technique that can be performed at the patient’s bedside, but it does not predict weaning outcome in COVID-19 patients. Further studies with specific endpoints need to be conducted before the role of DTF in weaning from ventilation can be defined, even in patients without COVID-19 respiratory failure.

## Data Availability

The datasets from this study are available from the corresponding author upon request.

## Abbreviations

DTF: Diaphragmatic thickening fraction
ARDS: Adult respiratory distress syndrome
RASS: Richmond Agitation-Sedation Scale
PEEP: Positive end-expiratory pressure
Tdi, Pi: Thickness during inspiration
Tdi, Ee: Thickness at end-expiration
SAPS2: Simplified Acute Physiology Score
